# Autophagy-Related Proteins GABARAP and LC3B Label Structures of Similar Size but Different Shape in Super-Resolution Imaging

**DOI:** 10.3390/molecules24091833

**Published:** 2019-05-13

**Authors:** Iman Abdollahzadeh, Johnny Hendriks, Julia L. Sanwald, Indra M. Simons, Silke Hoffmann, Oliver H. Weiergräber, Dieter Willbold, Thomas Gensch

**Affiliations:** 1 Institute of Complex Systems 4 (ICS-4, Cellular Biophysics), Forschungszentrum Jülich GmbH, Wilhelm-Johnen-Straße, 52428 Jülich, Germany; i.abdollahzadeh@fz-juelich.de (I.A.); jhendriks@upcmail.nl (J.H.); 2 Institute of Complex Systems 6 (ICS-6, Structural Biochemistry), Forschungszentrum Jülich GmbH, Wilhelm-Johnen-Straße, 52428 Jülich, Germany; si.hoffmann@fz-juelich.de (S.H.); d.willbold@fz-juelich.de (D.W.); 3 Institut für Physikalische Biologie, Heinrich-Heine-Universität Düsseldorf, Universitätsstraße 1, 40225 Düsseldorf, Germany; julia.sanwald@uni-duesseldorf.de (J.L.S.); indra.simons@uni-duesseldorf.de (I.M.S.)

**Keywords:** Atg8, autophagy, EYFP blinking, GABARAP, LC3B, shape distribution, single molecule localisation microscopy, SMLM, super-resolution

## Abstract

Subcellular structures containing autophagy-related proteins of the Atg8 protein family have been investigated with conventional wide-field fluorescence and single molecule localisation microscopy. Fusion proteins of GABARAP and LC3B, respectively, with EYFP were overexpressed in HEK293 cells. While size distributions of structures labelled by the two proteins were found to be similar, shape distributions appeared quite disparate, with EYFP-GABARAP favouring circular structures and elliptical structures being dominant for EYFP-LC3B. The latter also featured a nearly doubled fraction of U-shape structures. The experimental results point towards highly differential localisation of the two proteins, which appear to label structures representing distinct stages or even specific channels of vesicular trafficking pathways. Our data also demonstrate that the application of super-resolution techniques expands the possibilities of fluorescence-based methods in autophagy studies and in some cases can rectify conclusions obtained from conventional fluorescence microscopy with diffraction-limited resolution.

## 1. Introduction

Macroautophagy (hereafter autophagy) enables cells to replenish resources for energy metabolism and for anabolic reactions during periods of starvation, and to specifically dispose of large structures that are not amenable to proteasomal degradation. Correspondingly, autophagic cargo ranges from bulk cytosol to protein aggregates, damaged organelles, and even intracellular pathogens [1,2]. A hallmark of autophagy is the formation of double-membrane structures termed phagophores, which engulf cytoplasmic cargo and finally close to yield autophagosomes. The mature autophagosomes (several hundred nanometres in diameter) subsequently fuse with lysosomes, resulting in acidification and degradation of their contents by acid hydrolases. Genetic screening in yeast has led to the identification of more than 30 Atg genes, most of which are conserved in mammalian cells [3]. Among the corresponding proteins, Atg8 homologs serve different functionalities in autophagosome biogenesis and cargo recruitment. While in yeast only a single Atg8 is expressed, in mammalian cells the family has expanded into a number of paralogs assigned to either the GABA type A receptor-associated protein (GABARAP) or the microtubule associated protein 1 light chain 3 (MAP1LC3, hereafter LC3) subfamily [4].

Numerous studies have shown that proteins of both subfamilies are crucial for mammalian autophagy, and that they exert individual as well as subfamily- and family-specific functions [5,6]. Many of these activities relate to covalent attachment of Atg8 proteins to membrane lipids via their C-termini, enabling them to attract other components to autophagic membranes. These interactions are usually mediated by short linear motifs termed LIRs (LC3 interacting regions) in the target proteins, which bind to conserved hydrophobic pockets on the Atg8 protein surface [7]. For instance, Atg8 family members are well-known to participate in the recruitment of cargo to the concave face of expanding phagophores, exhibiting different specificities for so-called cargo receptors or the cargo molecules themselves [8]. Moreover, data from knockdown experiments and interaction studies point to a mechanistic role of GABARAP-type proteins in both early and late stages of autophagosome formation, involving interactions with components of the autophagy-initiating ULK complex and of the lysosome fusion machinery, respectively [9,10,11]. Members of the LC3 subfamily, on the other hand, appear to mostly support expansion of phagophores [9], but the respective molecular interactions are poorly defined and may involve lipids rather than proteins. Indeed, similar to their yeast ortholog, mammalian Atg8 proteins have been implicated in the regulation of membrane curvature and in vesicle adhesion and (hemi)fusion [12,13]; the mechanisms underlying these activities and their biological relevance, however, are only beginning to be unravelled. It is worth noting that autophagosome biogenesis has recently been observed to occur even in the absence of Atg8 proteins, albeit with reduced efficiency [14]. While this finding indicates substantial redundancy in the autophagy pathway, supporting fall-back operation even after loss of important components, it does not compromise the utility of proteins belonging to the Atg8 family—to the extent they are expressed—as markers of autophagic structures. Indeed, they are among the first proteins to be found on emerging phagophores shortly after nucleation, and continue to be present on the outer (convex) face at least until autophagosome closure (possibly longer), while on the inner (concave) face they are delivered for degradation together with cargo material [15].

Given the limited size of autophagy-related membrane structures (≈50 nm–1.5 µm), the spatial distribution of associated proteins can only imperfectly be resolved by conventional fluorescence microscopy. This method is subject to the diffraction limit of optical microscopy, which is represented by the Abbe criterion as d_x,y_ = λ/2NA, where d_x,y_ is the lateral resolution of the microscope, λ is the wavelength of the light, and NA is the numerical aperture of the optics. Hence, for a conventional microscope with NA ≈ 1, the Abbe limit for green light (λ ≈ 500 nm) is roughly 250 nm. The resolution improvement of fluorescence microscopy achieved with the development of super-resolution techniques enables the precise distribution of proteins of interest to be investigated [16]. One of the new methods is single-molecule localisation microscopy (SMLM), which relies on the accurate localisation (d_x,y_ on the order of 10–30 nm) of single fluorescent proteins based on the point spread function of their emitted photons, requiring fluorescence events to be recorded individually [17].

In the current study, we have used SMLM to investigate the spatial distribution of Atg8 proteins in mammalian cells, under autophagy-inducing conditions, using GABARAP and LC3B as representatives of the two subfamilies. At the same time, we aimed to evaluate the impact of the improved lateral resolution in SMLM, compared to conventional fluorescence microscopy, on the results of morphometric analysis. The geometry of fluorophore distribution is of key importance for the interpretation of results in a biological context because it directly relates to the type and stage of the underlying membranous structures. As a first step, we therefore focussed on the development of categories appropriate for systematic investigation of geometrical parameters (such as shape and size) of Atg8-positive structures. Statistical data analysis of SMLM images acquired from cells expressing enhanced yellow fluorescent protein (EYFP) fusion proteins revealed striking differences in shape distribution between GABARAP and LC3B. Moreover, the SMLM-based shape classification is at variance with the one obtained from conventional wide-field fluorescence microscopy, in particular if small- to medium-sized structures are considered.

## 2. Results

The shape and size distributions of EYFP-GABARAP and EYFP-LC3B containing structures, respectively, were investigated in fixed HEK293 cells, which were subjected to a standard protocol for enrichment of autophagic structures (starvation and block of autophagosome-lysosome fusion by application of bafilomycin A1) for 2 h right before fixation. For each overexpression, ten cells were selected for detailed evaluation. It was taken care that the selected cells were typical by not preferring cells with certain features, e.g., high (or low) number of fluorescent structures.

First, a fluorescence wide-field image (with diffraction-limited resolution) of the cell of interest was recorded using low excitation power. Subsequently, a pre-acquisition illumination (with 20- to 75-fold higher excitation power) of 20 to 120 s was performed, in which most of the EYFP molecules were photo-converted to metastable, non-fluorescent dark states. SMLM pictures of the EYFP-containing structures with super-resolution (i.e., resolution better than the diffraction limit) were obtained from a wide-field image series keeping the high excitation power mode and utilizing the enduring blinking behaviour of EYFP [18,19,20,21]. Measurement of this image series (typically 4000 frames with 50 ms observation time each) was started at the end of the pre-acquisition illumination period, when the remaining EYFP molecules in the fluorescent state were well separated and the maximum intensity in single frames equalled that known for our setup from other single molecule studies on EYFP. A computer program developed in our lab (SNSMIL; Shot Noise based Single Molecule Identification and Localisation [22]) was used to calculate a super-resolution SMLM picture from the image series.

**Figure 1:**
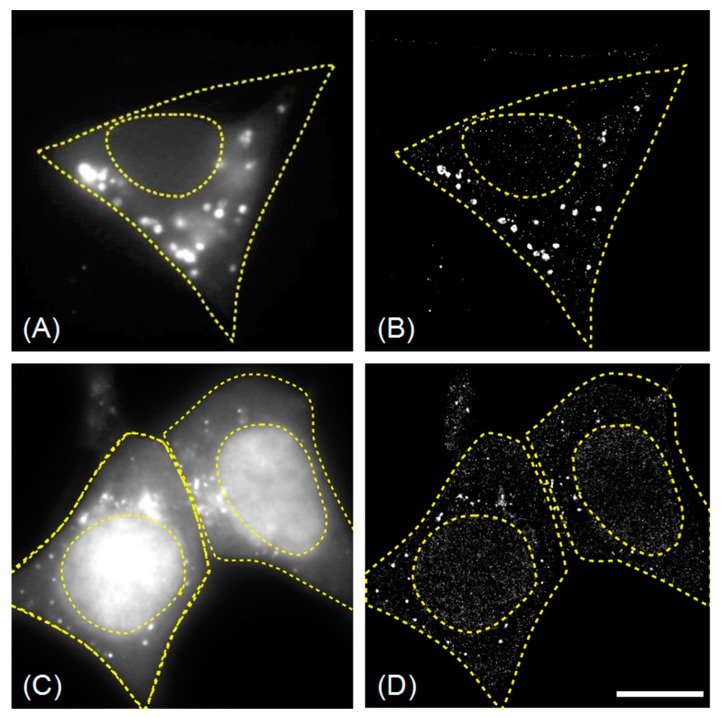
Wide-field (**A**,**C**) and SMLM (**B**,**D**) images of fixed HEK293 cells (starved and bafilomycin A1-treated) expressing EYFP-GABARAP (**A**,**B**), and EYFP-LC3B (**C**,**D**). In the wide-field fluorescence images only a few labelled structures are found (**A**: 22; **C**: 35 and 20 in the left and right cell, respectively), while in the corresponding super-resolution images (**B**,**D**) the numbers of EYFP-GABARAP containing structures (1640) and EYFP-LC3B containing structures (3100 and 1564) are almost two orders of magnitude larger. Scale bar (valid for **A**–**D**): 10 µm.

Representative examples of fixed HEK293 cells (starved and bafilomycin A1-treated) expressing EYFP-GABARAP and EYFP-LC3B, respectively, are given in Figure 1. The super-resolved SMLM images (Figure 1B,D) reveal a much higher total number of EYFP-GABARAP containing structures (75 times more) and EYFP-LC3B containing structures (89 and 78 times more for the left and right cell, respectively), compared to the corresponding wide-field images. A similar increase is found for all cells expressing one or the other fluorescent Atg8 construct. The number of EYFP-LC3B containing structures per cell (2898 ± 844) is significantly larger compared to the number of EYFP-GABARAP containing structures (1777 ± 356).

Notably, the subcellular distributions of the two overexpressed Atg8 proteins fused to EYFP are quite different: While EYFP-GABARAP is found mainly in the cytoplasm, EYFP-LC3B shows a higher concentration in the nucleus than in the cytoplasm. The larger labelled structures, however, are exclusively found in the cytoplasm for both proteins. These findings are in good agreement with data reported in literature [1,2,3,4,5,6]. Indeed, LC3B is thought to reside in the cell nucleus in an inactive acetylated form, which serves as a reservoir to be mobilised upon autophagy stimulation [23]; a different LC3 fraction associated with nuclear insulin receptor substrate 1 (IRS-1) has been suggested to attenuate autophagy in certain tumour cells [24]. GABARAP reservoirs, by contrast, have been identified on the ER and in the pericentriolar matrix [25], but not in the nucleus. Since the entire process of autophagosome biogenesis, maturation and degradation is known to take place outside the nucleus, we decided to focus on the cytoplasmic fraction of the fluorescently labelled objects for further analysis. Re-examination of the SMLM images under this premise yields much more similar values of 1550 ± 286 cytoplasmic EYFP-GABARAP containing structures (CS-EYFP-GABARAP) and 1813 ± 233 cytoplasmic EYFP-LC3B containing structures (CS-EYFP-LC3B), respectively (see Table 1). Thus, the before determined larger number of fluorescently labelled structures in EYFP-LC3B expressing cells, compared to EYFP-GABARAP expressing cells, is caused to a large extent by the nuclear protein fraction in the former case. One last remark to Figure 1C,D, i.e., wide-field and SMLM fluorescence images of EYFP-LC3B expressing cells, needs to be made. The fluorescence intensity contrast between nucleus and cytoplasm in Figure 1C is considerably larger when compared to the difference in numbers of fluorescent structures in those two areas of the cell. This can be explained by properties of the two detection methods, e.g., the larger focal depth of fluorescence wide-field imaging vs. single molecule detection or possible losses of single molecule detection when two many molecules emit in the same image.

### 2.1. Size Distributions of Cytoplasmic EYFP-GABARAP and EYFP-LC3B Containing Structures

**Figure 2:**
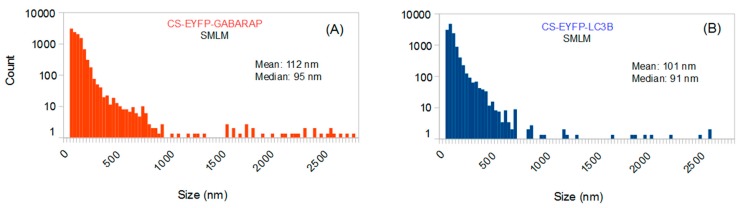
Size distributions (binning 32 nm) of all cytoplasmic, fluorescently labelled structures identified in the SMLM images of ten EYFP-GABARAP expressing (**A**) and ten EYFP-LC3B expressing HEK293 cells (**B**) under starvation and bafilomycin A1 treatment.

The size distributions of all CS-EYFP-GABARAP and CS-EYFP-LC3B are depicted in Figure 2. In general, they are very similar with mean and median slightly above and below 100 nm, respectively. The total number of CS-EYFP-LC3B per cell is only slightly larger than that of CS-EYFP-GABARAP. Notably, the vast majority of structures are smaller than the diffraction limit (ca. 200 nm for fluorescence imaging of EYFP).

For a more appropriate and meaningful comparison of the shape distributions of CS-EYFP-GABARAP and CS-EYFP-LC3B (Section 2.2), we considered it useful to divide the fluorescently labelled structures into two groups with respect to their size, namely small and large structures, for two reasons. First, there might exist a bias towards identifying circular shapes for small fluorescently labelled structures because of limited resolution and pixel size (16 nm in SMLM images). Second, large and small fluorescently labelled structures might well have different origins or functions and, as a consequence, also different shape distributions. We applied two different splitting values, 100 nm and 300 nm. The portion of fluorescently labelled structures larger than 300 nm will contain basically all structures whose shapes could be also classified with conventional fluorescence microscopy techniques like laser-scanning confocal or wide-field fluorescence microscopy. On the other hand, 100 nm is the upper size limit for most common intracellular vesicles in endocytic and secretory pathways. Table 1 reveals that the value of 100 nm splits the CS-EYFP-GABARAP in almost equally large groups, while splitting at 300 nm sees only 2% of the CS-EYFP-GABARAP in the group of the large structures and 98% belong to the small structures. For CS-EYFP-LC3B we find almost the same behaviour with about 40% and 3% of the structures larger than 100 nm and 300 nm, respectively.

Application of single molecule fluorescence imaging techniques might lead to differences with respect to conventional fluorescence microscopy for two reasons: (1) the different resolution, affecting the apparent shapes of visible objects, and (2) the different detection probability for structures as a function of their size. For a meaningful comparison of the information provided by the two methods, we generated sets of CS-EYFP-GABARAP and CS-EYFP-LC3B identifiable on wide-field fluorescence images of the cells (see Table 1).

**Figure 3:**
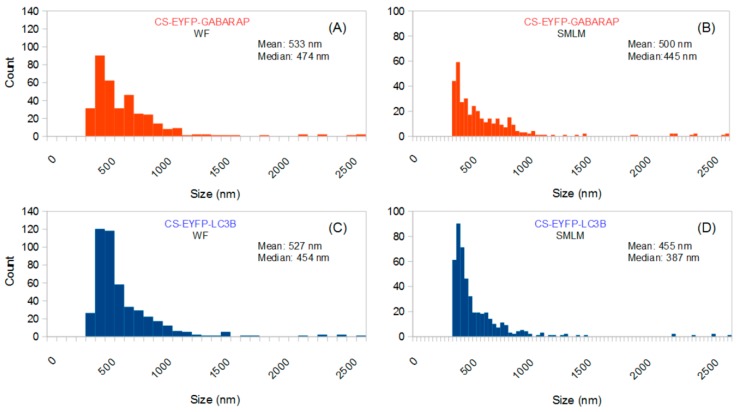
Size distributions of all conventionally selected fluorescently labelled structures (identified in wide-field images) of ten EYFP-GABARAP expressing (**A**,**B**) and ten EYFP-LC3B expressing fixed HEK293 cells (**C**,**D**) under starvation and bafilomycin A1 treatment, where size was determined in the wide-field fluorescence (**A**,**C**) and the SMLM images (**B**,**D**), respectively. Note the different binning used in **A**/**C** (80 nm) and **B**/**D** (32 nm), respectively, caused by the lower resolution in wide-field fluorescence compared to super-resolution microscopy.

The size distributions of these “conventionally selected” CS-EYFP-GABARAP and CS-EYFP-LC3B are given in Figure 3. As expected, the results of SMLM-based size analysis differ from those obtained when all structures found in the SMLM images are considered (compare Figure 2A with Figure 3B and Figure 2B with Figure 3D, respectively): No small structures (<300 nm) are found in the conventionally selected set, and mean and median are shifted towards higher values (between 455 and 533 nm). Interestingly, the size distributions of the conventionally selected CS-EYFP-GABARAP and CS-EYFP-LC3B, when determined in the conventional wide-field fluorescence images, were different from those using the respective super-resolution images (compare Figure 3A with Figure 3B and Figure 3C with Figure 3D): There is an increased number of structures smaller than 500 nm in the SMLM evaluation, although mean and median did not change dramatically.

### 2.2. Shape Classification

**Figure 4:**
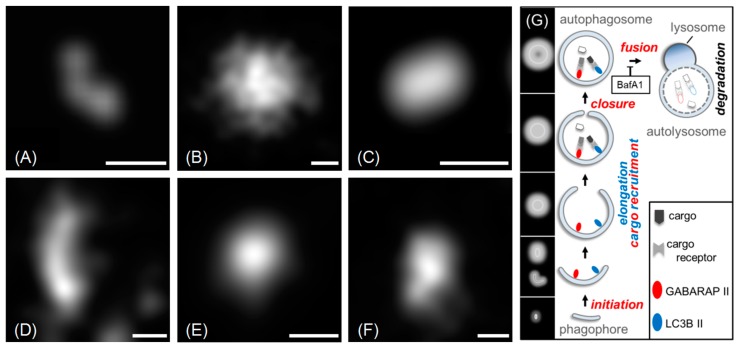
Examples for the three shape categories (**A**,**D**: U-shape; **B**,**E**: circle; **C**,**F**: ellipse). Structures in **A**, **B**, and **C** are from EYFP-GABARAP expressing cells, structures in **D**,**E**, and **F** are from EYFP-LC3B expressing cells (scale bars: 100 nm). In **G**, the presumed roles of GABARAP and LC3B at distinct stages of the autophagy pathway (phagophore initiation, elongation, closure, and fusion of the mature autophagosome with a lysosome to yield an autolysosome) are depicted. Steps that are assumed to require GABARAP and LC3B on the convex face of the isolation membrane (not drawn for clarity reasons) are highlighted in blue and red, respectively. As shown, GABARAP and LC3B can both link cargo materials to the concave face of the isolation membrane during selective autophagy in a cargo receptor-mediated manner. The various autophagic structures drawn as cross-sections are assigned to the respective 2D projections as anticipated in SMLM reconstructions (with a marked direction-dependence in the case of an early phagophore). Note, however, that SMLM cannot strictly distinguish between these membrane-bound autophagic organelles and other (vesicular or non-vesicular) structures populated by Atg8 proteins (see Discussion).

In the next step, we decided to group all fluorescently labelled cytoplasmic structures (within the size ranges defined above) according to their shapes. The shape classification used in this study has been limited to three geometrical categories, namely U-shape, circles and ellipses (abbreviated: u, c and e, respectively). This classification is based on the well-established mechanism of autophagosome formation (Figure 4G and [6,26,27]), where these three shape categories reflect all possible autophagic structures. In the beginning of autophagosome formation, a small double bilayer (i.e., a phagophore) grows around the cargo and may appear as a U-shape structure (when viewed from the side) or as a relatively small circular (when viewed along is longitudinal axis) or elliptical object (when viewed at intermediate angles) in the two-dimensional SMLM imaging mode. During the elongation phase, the phagophore geometry is approaching a half-moon or elliptical shape. Once the autophagosome is mature, its shape will be very similar to a sphere, i.e., a circle in our 2D-projection SMLM imaging mode, except for very large and asymmetrical cargo. In Figure 4, typical examples for the three categories of EYFP-GABARAP and EYFP-LC3B containing structures, respectively, from SMLM images of fixed HEK293 cells are depicted.

#### 2.2.1. Shape Distributions of Cytoplasmic EYFP-GABARAP and EYFP-LC3B Containing Structures Selected in Super-Resolution Fluorescence Microscopy Images

**Figure 5:**
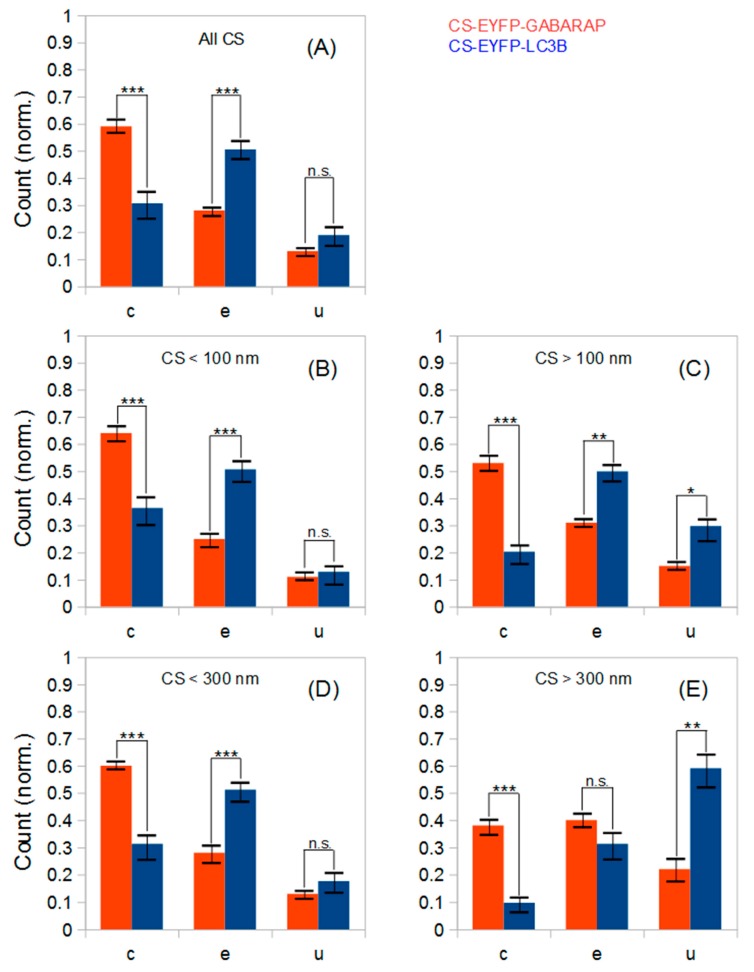
Shape analysis of all cytoplasmic fluorescent structures observed in SMLM images of ten HEK293 cells expressing either EYFP-GABARAP (red) or EYFP-LC3B (blue) under starvation and bafilomycin A1 treatment. (**A**) All CS-EYFP-GABARAP (15501) and CS-EYFP-LC3B (18129); (**B**) CS-EYFP-GABARAP (8009) and CS-EYFP-LC3B (11579) smaller than 100 nm; (**C**) CS-EYFP-GABARAP (7492) nd CS-EYFP-LC3B (6550) larger than 100 nm; (**D**) CS-EYFP-GABARAP (15153) and CS-EYFP-LC3B (17643) smaller than 300 nm; (**E**) CS-EYFP-GABARAP (348) and CS-EYFP-LC3B (486) larger than 300 nm. Error bars represent standard error of the mean. Statistical significance is represented as *P* ≤ 0.01 (∗∗∗); *P* ≤ 0.05 (∗∗), and *P* ≤ 0.1 (∗) from two-tailed t-tests (n.s., not significant). c, e and u stands for circles, ellipses and U-shapes.

Classification of all CS-EYFP-GABARAP in the SMLM images led to a unique shape distribution similarly found in all ten fixed HEK293 cells analysed (Figure 5). The number of CS-EYFP-GABARAP per cell varied between 350 and 2300 with a total number of 15501 in all ten cells (Table 1). To better compare the shape distributions of CS-EYFP-GABARAP, we considered it useful to divide the CS-EYFP-GABARAP in two groups, small and large structures, with two different splitting values, 100 and 300 nm, respectively (see paragraph 2.1). The shape distributions in the five size cases (all structures, and structures smaller/larger than 100 nm/300 nm) can be generally described as follows: The majority of CS-EYFP-GABARAP appear as circles, fewer structures as ellipses and a minor fraction shows U-shape (only for CS-EYFP-GABARAP larger than 300 nm, circles and ellipses have similar occurrence; Figure 5E). U-shape structures show the lowest percentage among CS-EYFP- GABARAP (only 9 to 23%) with the highest value in the group of the largest CS-EYFP-GABARAP (>300 nm).

Classification of all CS-EYFP-LC3B occurred in the same way as described above for CS-EYFP-GABARAP (applying again the separation values of 100 nm and 300 nm, respectively). The total number of CS-EYFP-LC3B in the ten analysed transfected HEK293 cells amounted to 18129 and was hence slightly (ca. 20%) larger compared to the experiments with CS-EYFP-GABARAP. Yet, comparison of the total number of labelled structures in an experiment based on overexpression of proteins is not useful, since a number of experimental parameters may vary in transient transfections (e.g., quality of DNA, efficiency of plasmid uptake, yield of chromophore maturation), preventing the reproducibility of absolute protein numbers. The shape distributions of structures labelled with Atg8-family proteins, on the other hand, will reflect specific properties and biological functions of GABARAP and LC3B, respectively, as long as expression levels are not too high.

The five shape distributions of CS-EYFP-LC3B are plotted in Figure 5 next to the corresponding ones of CS-EYFP-GABARAP, and direct comparison immediately reveals that populations of structures labelled by EYFP-LC3B and EYFP-GABARAP differ from one another. For all five categories of CS-EYFP-LC3B, ellipses constitute the major fraction (31–51%), while circles—the major fraction for CS-EYFP-GABARAP—were only the second most prevalent for total and the small-size groups of CS-EYFP-LC3B (ca. 30%) and even the minor fraction for the large-size groups of CS-EYFP-LC3B (10–20%). U-shape objects among the CS-EYFP-LC3B were found to be present in higher relative amounts (13–59%) compared to CS-EYFP-GABARAP, being the most abundant shape for CS-EYFP-LC3B larger than 300 nm.

The total number of analysed structures is high and the shape distributions for CS-EYFP-GABARAP and CS-EYFP-LC3B, respectively, appear visually different, especially for the large-size structures. The number of cells analysed (10 for each protein), however, is only moderate. Nevertheless, we performed two-tailed t-tests to survey whether the differences in the relative abundances of circles, ellipses and U-shapes are statistically significant. The results are given in numbers in Table 2, and most of them are also shown graphically in Figure 5.

The five shape distributions of CS-EYFP-GABARAP are all statistically significantly different from the corresponding distributions of CS-EYFP-LC3B with at least one P-value smaller than 0.01 and a second one smaller than 0.05. The higher amount of U-shapes for larger cytoplasmic structures containing EYFP-LC3B compared to those containing EYFP-GABARAP is significant too–albeit at a weaker level. Thus, though the number of investigated cells is only moderately high (due to the time-consuming and elaborate size and shape analysis), the shape differences between the structures labelled by the two proteins are highly relevant.

#### 2.2.2. Shape Distributions of Cytoplasmic EYFP-GABARAP and EYFP-LC3B Containing Structures Selected in Wide-Field Fluorescence Microscopy Images

Most fluorescence microscopy studies using Atg8-family proteins fused to fluorescent proteins until now have been carried out using fluorescence microscopy methods with diffraction-limited resolution. As pointed out in Section 2.1, we performed a “hybrid” analysis of our data set, where we carried out a conventional selection of CS-EYFP-GABARAP and CS-EYFP-LC3B and determined their size distribution via conventional as well as super-resolution imaging (Figure 3). As expected, the number of cytosolic fluorescently labelled structures detected by “conventional selection” was found largely reduced and is well below 50 per cell. The improved spatial resolution in the SMLM compared to wide-field fluorescence images, however, unearthed an even more serious observation. The result of the shape classification for one and the same structure can be different in a wide-field fluorescence image and its corresponding SMLM image, respectively.

**Figure 6:**
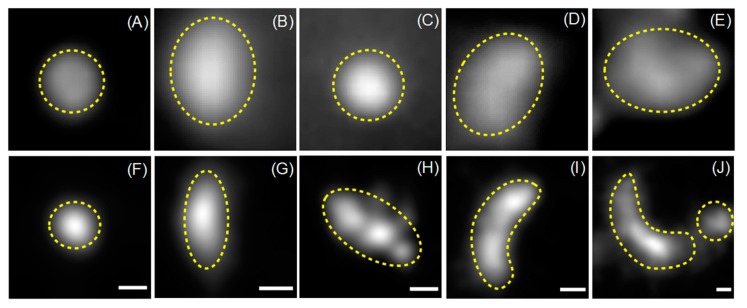
Wide-field fluorescence (**A**–**E**) and SMLM images (**F**–**J**) of five CS-EYFP-LC3B that were identified on the basis of the wide-field image (scale bars: 100 nm).

To demonstrate this issue, wide-field and super-resolution fluorescence images of five conventionally selected CS-EYFP-LC3B are depicted in Figure 6 (similar examples can be found for CS-EYFP-GABARAP). The structures in A and C are circles, while B, D and E fall into the ellipse category when judged by the wide-field fluorescence images. This simple picture changes when examining the fluorescent structures in the respective SMLM images. While the structures in A (circle) and B (ellipse) appears to have the same shape in super-resolution (compare with F and G, respectively), the other three structures have a different shape when imaged and analysed with higher spatial resolution. The ellipse in D turns into a U-shape in I, while the circle in C resolves into an inhomogeneous ellipse in H. The ellipse in E even appears to be clearly two objects in J, a U-shape and a circle.

**Figure 7:**
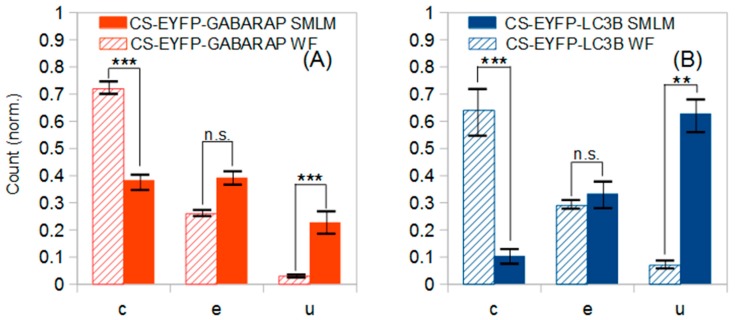
Shape distributions of fluorescently labelled cytoplasmic structures identified in the wide-field images of ten EYFP-GABARAP expressing and ten EYFP-LC3B expressing HEK293 cells, respectively, under starvation and bafilomycin A1 treatment. (**A**) Conventionally selected CS-EYFP-GABARAP (348) classified in wide-field fluorescence (hatched bars) and the same structures classified in the respective SMLM (full bars) images; (**B**) Conventionally selected CS-EYFP-LC3B (486) classified in wide-field fluorescence (hatched bars) and the same structures classified in the respective SMLM (full bars) images. Error bars represent standard error of the mean. Statistical significance is represented as *P* ≤ 0.01 (∗∗∗); *P* ≤ 0.05 (∗∗), and *P* ≤ 0.1 (∗) from two-tailed t-tests (n.s., not significant). c, e and u stands for circles, ellipses and U-shapes.

We analysed the shapes of the “conventionally selected” CS-EYFP-GABARAP and CS-EYFP-LC3B and made a striking observation. For both Atg8 proteins we find a significant difference of the shape distribution dependent on whether the shape was classified in wide-field or super-resolution fluorescence images (Figure 7). U-shapes are very rare (<3%) for CS-EYFP-GABARAP in wide-field fluorescence and grow to ca. 20% in super-resolution images. The relative amount of elliptical shape increases from below 15% to almost 40% on the expense of the circular shape, whose percentage drops from above 80% to 55% (Figure 7A). The change of shape distribution is even more pronounced for CS-EYFP-LC3B when comparing wide-field and super-resolution fluorescence imaging (Figure 7B). Here, circular shapes turn from the dominant fraction (75%) in wide-field fluorescence to the minor fraction (ca. 15%) in super-resolution fluorescence, while the percentage of elliptical shape more than doubles and U-shape grows from less than 5% to almost 40%. For both proteins, the relative abundances for circles and U-shapes are statistically highly significantly different as can be seen in Figure 7 and Table 2.

One more relevant observation of more general nature has to be stated, namely that the shape distributions of the structures containing the two Atg8 proteins appear very similar upon examination in conventional fluorescence microscopy with diffraction-limited resolution (none of the three shape categories has a statistically different relative abundance, see Table 2), but are rather different when shape is judged in super-resolution SMLM fluorescence microscopy. A direct comparison is depicted in Figure 7, since for both proteins the group of cytoplasmic fluorescent structures identified in wide-field images is identical to the group of cytoplasmic fluorescent structures larger than 300 nm identified in SMLM images (fractions of both circles and U-shapes are significantly different). Our data point towards involvement of GABARAP and LC3B in different stages of autophagosome biogenesis or participation in further, non-autophagy related processes. But this difference would have been overlooked when using conventional fluorescence microscopy methods.

## 3. Discussion

The introduction of optical microscopes has marked a revolution in the biological sciences since it enabled the cellular structure of organisms to be directly viewed for the first time [28]. After several centuries, and notwithstanding numerous technical improvements, the basic principle of using lenses to generate magnified views of samples is still widely utilised and continues to provide valuable insight into biological matter. An important complement has been the development of fluorescent tags, which allowed structures of interest to be specifically labelled in both fixed and live cells and which integrated nicely with existing microscopic technology.

It therefore comes as no surprise that fluorescence-enhanced light microscopy has been a major visualisation tool in autophagy research [29]. Autophagic organelles are complex membrane structures undergoing shape transformations during their life cycle, and both morphogenesis and functionality of these membranes are thought to be controlled by associated proteins. Hence, specific microscopic detection of autophagy-related proteins may not only provide hints at their biological functions but can also help to visualise the underlying organelle as a whole, provided that the marker is indeed distributed throughout the structure of interest. Atg8 family proteins are thought to largely meet this requirement; while in-vitro experiments revealed a certain preference for curved membranes [12], enrichment at the edges of expanding phagophores, e.g., has not been demonstrated thus far. Over almost two decades, Atg8 imaging has contributed to a huge body of literature, the vast majority of which, however, has been compromised by the diffraction limit of conventional microscopy. Super-resolution techniques, which basically extract highly precise positional information from microscopic images which per se are diffraction-limited, help to alleviate this shortcoming and are starting to contribute new insight into the autophagy process.

In the current study, we have used SMLM to revisit several intriguing questions in the field: (1) the distribution of sizes and shapes of structures labelled by Atg8 family proteins, resulting from a non-synchronous evolution of numerous individual objects, and (2) the differential localisation of GABARAP and LC3B (representing the two Atg8 subfamilies), which is closely related to their functions on a molecular level.

Our data demonstrate that application of SMLM has the potential to provide superior results in comparison with conventional wide-field microscopy, in terms of both completeness of observations (Figure 1 and Figure 2) and wealth of associated information (Figure 5 and Figure 6). These criteria bear obvious relation with the nominal spatial resolution of the respective images. Observational completeness can be defined as the fraction of the items of interest that can be detected using the method in question. In our hands, SMLM captures about 50 times the number of EYFP-labelled structures found via wide-field microscopy; comprehensive analysis of the size distributions revealed this difference to be mainly due to a vast number of smaller structures (those with diameters < 300 nm) going unnoticed on the wide-field images (Table 1, Figure 3). This is a huge limitation given that a significant portion of autophagy-related structures should fall into this size range, including early isolation membranes and even smaller-sized mature autophagosomes. The advantages of super-resolution microscopy in terms of information content are supported by the observation that even a set of larger structures, which are readily visible on both wide-field and SMLM images, yields quite different data depending on the method used for analysis. In addition to a distortion of size distributions at lower resolution (Figure 3), we find large effects on the assignment of shapes (Figure 6 and Figure 7). The latter illustrates what we consider the most significant corollary of the current study: While structures with dimensions on the order of (or slightly above) the diffraction limit may be readily detectable by conventional microscopy, the information extracted from such images should be treated with caution because sizes and shapes may be biased. Autophagy constitutes an instructive example of a process in which the morphology of organelles directly reflects their functional state, and misinterpretation of, e.g., a U-shape structure (commonly assigned to an early phagophore viewed from the side) as an elliptical object (usually assigned to a late phagophore or autophagosome) may affect biological conclusions drawn from experiments.

Besides these methodological aspects, our SMLM analysis using EYFP fusion proteins revealed important differences between the two Atg8 orthologs investigated. In particular, the shape distributions of the respective labelled structures are quite disparate, indicating differential (but possibly overlapping) localisation: GABARAP and LC3B may appear on phagophores at different stages of their evolution, but could also support distinct autophagic channels or participate in non-autophagy-related pathways. These considerations are well-supported by current evidence. For instance, experiments in which entire Atg8 subfamilies have been knocked down or knocked out in cultured cells suggested that LC3 proteins were mostly required for phagophore expansion, whereas GABARAP proteins acted at a later stage, such as maturation, closure, or autophagosome-lysosome fusion [9,14]. Such a division of tasks would seem consistent with the preponderance of circular and elliptical shapes in our SMLM images after overexpression of GABARAP and LC3B, respectively. It is interesting to note that this correlation can even be replicated in vitro: Vesicles coated with GABARAP tend to fuse into approximately spherical structures whereas LC3B coupling yields more elongated shapes [13]. Regarding their functions in cargo recruitment, members of the Atg8 family are well-established to differ in their affinities towards target structures, often with marked subfamily specificity [7], supporting the idea that the prevalence of different Atg8 orthologs on individual phagophores or autophagosomes may be modulated by the type of substrate. It is also worth noting that enrichment of Atg8 proteins in punctate objects does not necessarily signify phagophores or autophagosomes. The centrosomal pool of GABARAP, e.g., which is thought to play a critical role in autophagosome biogenesis, presumably consists of non-lipidated protein [25], and indeed, neither the centrosomal matrix nor the centriolar satellites shuttling GABARAP along microtubules contain membrane vesicles. Similarly, nuclear association of LC3 with IRS-1 leads to the formation of layered clusters not involving biological membranes [24]. Mammalian Atg8 proteins have also been found to associate with IRGM (immunity-related GTPase M) and the Q_a_-SNARE syntaxin-17 (Stx17) in large protein complexes (so-called autophagosome recognition particles, ARPs) which deliver Stx17 to mature autophagosomes, thus enabling fusion with lysosomes. [30]. Again, these structures are assumed to be non-membranous, with IRGM shielding the transmembrane domain of Stx17. Finally, both GABARAP and LC3B participate in cellular processes that are unrelated to autophagy but do involve vesicular structures. Prominent examples include trafficking of vesicles carrying transmembrane receptors towards the plasma membrane, which is typically mediated by GABARAP subfamily proteins [5], and LC3-associated phagocytosis, which is usually engaged if membrane-wrapped extrinsic cargo is to be degraded [31]. A more general function in cellular signalling has emerged for GABARAP-type proteins, which are able to recruit a ubiquitin ligase targeting the RAC1-specific guanine nucleotide exchange factor TIAM1 (T-lymphoma invasion and metastasis-inducing protein 1). This process has been suggested to occur on nonautophagic membranes, although mechanistic connections to autophagy regulation may exist [32]. Based on these considerations, it seems very likely that a certain fraction of the objects labelled by EYFP-fused Atg8 proteins actually constitute non-membranous autophagic or membranous non-autophagic structures, and thus do not represent phagophores or autophagosomes. The abundance of fluorescently labelled particles in the sub-100 nm range, for instance, may be explained to a large part by non-vesicular structures like centriolar satellites or ARPs. While protein complexes of this size are clearly resolved in our SMLM images, they only contribute to a diffuse background in conventional diffraction-limited microscopy, preventing their differentiation from the cytosolic Atg8 pool. Despite the significant gain in spatial information provided by super-resolution fluorescence imaging, unambiguous assignment of structures to specific pathways or even intermediates thereof still requires secondary labelling for a plethora of markers, and will be the subject of future work.

In order to assess the localisation of GABARAP and LC3B, we have resorted to transient overexpression of fluorescent fusion proteins, which is part of the standard toolkit in cell biology research. In comparison to immunolabelling of endogenous protein, this strategy ensures decent signal strength and excellent specificity of detection, but comes with the downside of potentially non-specific localisation of overexpressed protein. The latter might accumulate at sites which are not significantly populated in parent cells, and even at physiological locations pathways may suffer from an overload of protein as well as the presence of the fusion partner. The strength of nuclear staining we observed with EYFP-LC3B even after autophagy stimulation may indicate such an effect of overexpression since it exceeds what has been described previously for endogenous LC3 detected by immunofluorescence. On the other hand, the numbers of cytoplasmic structures populated with EYFP-GABARAP and EYFP-LC3B are quite similar despite the fact that total cytoplasmic fluorescence (representing the abundance of the fusion protein) differs by a factor of five; this suggests that the overall activity of the respective pathways is at most moderately affected by Atg8 protein overexpression, thus supporting the validity of the approach. Development of cell lines stably expressing fluorescent fusion proteins under the control of endogenous promoters will be instrumental to avoid artefacts caused by protein overload while retaining the specificity of detection in both conventional and super-resolution imaging modes.

## 4. Materials and Methods

### 4.1. Eukaryotic Plasmids

The gene for GABARAP was subcloned from a GST-GABARAP-fusion plasmid (Addgene plasmid #73948 [33], Addgene, Watertown, MA, USA) by PCR amplification into the XhoI and BamHI sites of peYFP-C1 (Clontech, Mountain View, CA, USA), yielding peYFP-C1/GABARAP [34]. The fluorescent variant of LC3B was generated analogously, starting from a GST-fusion plasmid (Addgene plasmid #73949 [33], Addgene) and yielding peYFP-C1/LC3B.

### 4.2. Cell Culture and Transfection

Human embryonic kidney 293 (HEK293 [35]; Leibniz-Institute DSMZ–German Collection of Microorganisms and Cell Cultures, Braunschweig, Germany) cells were cultivated at 37 °C in a humidified incubator at 5% CO_2_ in Dulbecco’s Modified Eagle’s Medium (DMEM, Cat. No. D5796, Sigma-Aldrich, Munich, Germany) supplemented with 1% penicillin/streptomycin (P/S, Sigma-Aldrich) and 10% Fetal Calf Serum (FCS, Sigma-Aldrich). For transient transfection with EYFP-GABARAP or EYFP-LC3B constructs, 6 × 10^5^ HEK293 cells were seeded into a 6-well culture plate (Cat. No. 10062-892, VWR, Randor, PA, USA) containing DMEM with 10% FCS and 1% P/S. On the next day, transfection with 1.2 μg total DNA was performed using Polyfect (Cat. No. 301107, QIAGEN, Hilden, Germany) according to the manufacturer’s instructions. The following day, 4–5 × 10^4^ of the transfected cells were seeded into a fibronectin (Sigma-Aldrich) coated µ-dish (Cat. No. 81158, ibidi, Martinsried, Germany) containing DMEM with 10% FCS and 1% P/S and were cultivated for another day in the incubator.

### 4.3. Starvation

Transfected cells were starved with Hank’s Balanced Salt Solution (HBSS, Cat. No. 14025050, Thermo Fisher Scientific, Waltham, MA, USA). For accumulation of autophagic structures in cells, 100 nM bafilomycin A1 (CAS No. 0088899552, Merck KGaA, Darmstadt, Germany) was used as an autophagosome-lysosome fusion blocking agent along with HBSS. Incubation took place at 37 °C and 5% CO_2_ for 2 h.

### 4.4. Fixation

Since autophagic structures are supposed to be connected to cytoskeleton elements, a fixation procedure was used that minimises alterations to cytoskeleton components [36]. The cells were incubated in cytoskeleton buffer (phosphate-buffered saline (PBS, 137 mM NaCl, 2.7 mM KCl, 1.8 mM KH_2_PO_4_, 10 mM Na_2_HPO_4_, pH 7.4) containing 4 mM EGTA) for 5 min at 37 °C. Subsequently, cytoskeleton buffer was replaced with fixation solution (4% (*w/v*) paraformaldehyde in cytoskeleton buffer). Fixation took place at room temperature for 10–15 min. After that, cells were rinsed three times with 1 M glycine in PBS and two times with PBS.

### 4.5. Imaging

For visualisation of fluorescent structures with spatial resolution of the order of 20–30 nm, a home-built SMLM microscope was used [21,37,38]. For SMLM imaging of EYFP-GABARAP and EYFP-LC3B, cells were kept in PBS (pH 7.4). Cells, stored at 4 °C after fixation, were accommodated to room temperature for about 30 min before imaging, because otherwise the recorded image showed lateral and focal drift of the order of several hundreds of nanometres. EYFP was excited with the 514 nm line of an Ar+ laser (Innova 70C, Coherent, Santa Clara, CA, USA). In single-color SMLM experiments with either EYFP-GABARAP or EYFP-LC3B containing structures, the fluorescent protein was first bleached for 10–60 s with 75% laser power until single fluorescent EYFP molecules could be observed in the field of view. The acquired number of single molecule images amounted to 4000–8000. The camera exposure time was set to 50 ms. Image analysis and super-resolution reconstruction were performed with the SNSMIL software, which has been described elsewhere [22]. In this study, the SNSMIL quality parameter was set to 1 to ensure maximum single molecule identification efficiency. The uncertainties of x- and y-position of single EYFP-GABARAP and EYFP-LC3B molecules in the image plane were determined as 38 nm and 27 nm, respectively. With such experimental conditions, the practical resolution of structures in the SMLM images is well below 50 nm.

### 4.6. Shape and Size Analysis

Evaluation of shape and size of EYFP-GABARAP and EYFP-LC3B containing cytoplasmic structures was performed with the use of ImageJ [39] in a semi-automated approach. The reconstructed super-resolved image was first converted to a binary image containing only values of 0 or 1. A lower size cut-off must be applied in order to discard very small structures originating from isolated EYFP emitters not linked to EYFP-GABARAP or EYFP-LC3B containing structures, autofluorescence, or noise. We chose 50 nm, well above the positional uncertainty for single molecule emitters in our setup. In the case of asymmetric objects, the longest dimension of the structure had to fall in this size range. Shape classification of CS-EYFP-GABARAP and CS-EYFP-LC3B was performed by visual inspection by the experimenter based on three different geometrical patterns, named circles (c), ellipses (e), and U-shapes (u; see paragraph 2.2 and Figure 4; compare also [40]). The statistical significance of differences in relative abundances of shapes was assessed with the two-tailed t-test function of LibreOffice Calc (Version 5.2; The Document Foundation, Berlin, Germany).

### 4.7. Selection of Cytoplasmic EYFP-GABARAP and EYFP-LC3B Containing Structures

In order to identify the part of cytoplasmic EYFP-GABARAP and EYFP-LC3B containing structures, a contour around the nucleus was drawn in the transmission (bright field) image of the cell. This contour was transferred to the corresponding SMLM image and the structures within the contour were registered as nucleus-related structures. The remainder of the detected fluorescent structures in the super-resolution image was considered cytoplasmic (yielding the subsets CS-EYFP-GABARAP and CS-EYFP-LC3B) and further analysed.

## Figures and Tables

**Table 1:** Number of cytoplasmic structures containing EYFP-GABARAP and EYFP-LC3B identified in SMLM images. Counts are given for the entire size range considered (50 nm–2.8 µm) as well as for two sub-ranges (split alternatively at 100 nm or at 300 nm). For comparison, the numbers of cytoplasmic EYFP-GABARAP and EYFP-LC3B containing structures identified in the corresponding wide-field fluorescence images (named “conventional selection”) are also given.

Overexpressed Protein	Size Category	Number of Structures	Fraction of Structures (%)
EYFP-GABARAP	50 nm–2.8 µm	15,501	100
	50 nm–100 nm	8009	51.66
	100 nm–2.8 µm	7492	48.33
	50 nm–300 nm	15,153	97.75
	300 nm–2.8 µm	348	2.25
	Conventional selection	348	
EYFP-LC3B	50 nm–2.8 µm	18,129	100
	50 nm–100 nm	11,579	63.87
	100 nm–2.8 µm	6550	36.13
	50 nm–300 nm	17,643	97.32
	300 nm–2.8 µm	486	2.68
	Conventional selection	486	

**Table 2:** P-values from two-tailed t-tests to assess the statistical significance of differences in the shape distributions (c, circles; e, ellipses; u, U-shapes) of (i) CS-EYFP-GABARAP vs. CS-EYFP-LC3B (various size classes), (ii) different size classes of CS-EYFP-GABARAP, (iii) different size classes of CS-EYFP-LC3B, and (iv) CS-EYFP-GABARAP or CS-EYFP-LC3B structures classified in wide-field vs. SMLM. P-values are indicated by shading and typeface (≤0.01, dark grey and bold face; ≤0.05, medium grey and bold face; ≤0.1, no shading and regular face) for ease of orientation.

	c	e	u
CS-EYFP-GABARAP vs. CS-EYFP-LC3B (50 nm–2.8 μm)	**0.0026**	**0.00050**	0.21
CS-EYFP-GABARAP vs. CS-EYFP-LC3B (< 100 nm)	**0.010**	**0.0053**	0.66
CS-EYFP-GABARAP vs. CS-EYFP-LC3B (> 100 nm)	**0.000026**	**0.019**	**0.10**
CS-EYFP-GABARAP vs. CS-EYFP-LC3B (< 300 nm)	**0.0024**	**0.00035**	0.29
CS-EYFP-GABARAP vs. CS-EYFP-LC3B (> 300 nm)	**0.000023**	0.49	**0.04**
CS-EYFP-GABARAP: CS < 100 nm vs. CS > 100 nm	0.29	0.54	0.20
CS-EYFP-GABARAP: CS < 300 nm vs. CS > 300 nm	**0.0042**	**0.02**	0.23
CS-EYFP-LC3B: CS < 100 nm vs. CS > 100 nm	**0.09**	0.71	**0.04**
CS-EYFP-LC3B: CS < 300 nm vs. CS > 300 nm	**0.03**	**0.08**	**0.02**
CS-EYFP-GABARAP: WF vs. SMLM	**0.000010**	0.50	**0.0018**
CS-EYFP-LC3B:WF vs. SMLM	**0.000010**	0.70	**0.01**
